# Ohio State Dental Society

**Published:** 1897-01

**Authors:** 


### EDITORIAL.

### Ohio State Dental Society.

The annual meeting of this body was held at the Neal House in Columbus, December 1st, 2nd and 3rd. A very interesting and varied programme was presented for the attention and consideration of the Society. The programme was completely carried out as arranged, with the exception of the reading of one paper, the writer of which was detained by sickness. Every paper read was of unusual interest and quite freely discussed. One very encouraging feature of the meeting was, that a large proportion of the papers read were prepared by comparatively young members of the profession. Among the papers presented was one by Dr. George H. Wilson, of Cleveland, on "Dental Legislation," in which this subject was quite fully discussed. The defects in the Ohio law were pointed out, as also the amendments that should be made in the same to render the best service of such a law to the profession and public.

The subject, "Cataphoresis," was quite fully presented in four or five papers. The first, "A Summary of Experience with Cataphoresis," by Dr. L. L. Barbour, of Toledo; second, "The Galvanic Current," by Dr. H. G. Husted, of Oberlin; third, "Some Reasons Why We Fail with Cataphoresis," by Dr. Wm. H. Hirsch, of Piqua. The fourth paper on this subject was a very extensive one, by Dr. M. A. Price, of Cleveland, on "The Management of the Electric Current in Cataphoresis," and the fifth paper, "Bleaching by Catophoresis," with clinic, was presented by Dr. Henry Barnes, of Cleveland. These papers were read successively, and then the whole subject thoroughly discussed by the members. Many very interesting, and some new, points were brought out in the papers and discussions. The various methods of employing the electric current for this work were more fully elaborated than they, perhaps, have ever been at any similar meeting. Quite a variety of views and experiences were given in both papers and discussions. But few claimed universal success for the cataphoric process. Not all, by any means, claimed for it general success; some claimed that it was no more efficient in the treatment of sensitive dentine than other methods that have been employed extensively. A few of the members who had extended their investigations further than most of the others, claimed great efficiency in anæsthetizing dental pulps for treatment or removal. From quite a number of experiments reported it would seem that this will prove of quite important use in this process.

A paper on "Empyemia of the Maxillary Sinus" was given by Dr. J. T. Stephan, of Cleveland, which elicited an extensive and very interesting discussion. "Bacteria of the Mouth" was presented, in an excellent paper, by Dr. C. T. Whinnery, of Toledo, also a paper on "Imagination," by Dr. J. S. Cassidy, of Covington. "Antiseptics and Disinfectants in Office Practice" was presented in a paper by Dr. H. B. Bartleson, of Columbus.

As thus far outlined, only a part of what was done at the meeting is given. There was a larger attendance than has been for years, and a larger number of new members admitted than have been at any one time since the reorganization of the Society. There was also more enthusiasm than has for a long time been shown. Judging from the indications prevalent at this meeting the Society has before it a very bright future, which, we trust, will be fully realized.

